# The Structure and Performance of Short Glass Fiber/High-Density Polyethylene/Polypropylene Composite Pipes Extruded Using a Shearing–Drawing Compound Stress Field

**DOI:** 10.3390/ma12081323

**Published:** 2019-04-23

**Authors:** Yi Yuan, Changdong Liu, Meina Huang

**Affiliations:** 1 Chongqing Key Laboratory of Manufacturing Equipment Mechanism Design and Control, Chongqing 400067, China; meinahuang1982@ctbu.edu.cn; 2 Department of Mechanical Design and Manufacturing, College of Mechanical Engineering, Chongqing Technology and Business University, Chongqing 400067, China; 3 Engineering Research Center for Waste Oil Recovery Technology and Equipment of Education, Chongqing 400067, China; 4 Dongfeng Sokon Motor Co., Ltd., Chongqing 405321, China; liuchangd@163.com

**Keywords:** shearing–drawing compound stress field, shear rotating speed, shear inducement, two-dimensional self-reinforcement pipe-extrusion, short glass fiber/high-density polyethylene/polypropylene composite pipe

## Abstract

Glass fiber reinforced polyolefin composite materials have many advantages regarding their performance and have been widely used in many fields. However, there are few reports on the simultaneously bidirectional self-enhancement of glass fiber reinforced polyethylene/polypropylene composite pipe. To self-reinforce the pipe’s circular and axial properties simultaneously, short glass fiber reinforced high-density polyethylene/polypropylene (SGF/HDPE/PP) pipes were extruded using a shearing–drawing two-dimensional compound stress field pipe-extrusion device. The effects of the rotating speed of the rotating shear sleeve on the orientation, heat behavior, microstructure, and tensile strength of the pipe were investigated in this paper. The microstructure was observed using scanning electron microscopy (SEM), and the crystal diffraction was analyzed using a polycrystalline X-ray diffractometer (WAXD), the heat behavior was measured using a differential scanning calorimeter (DSC), and the tensile strength was tested using a universal electronic tensile testing machine. The results showed that the shear induction effect induced by the shear rotating promoted the formation of the oriented structure of the crystal plate and SGFs along the circular and axial directions of the pipe simultaneously. Furthermore, it increased the crystallinity of the system, and self-improved the pipe’s circular and axial tensile strength at the same time.

## 1. Introduction

Glass fiber reinforced polyolefin composite materials have many advantages such as a high elastic modulus, good mechanical properties, good dimensional stability, excellent electrical performance, short molding cycle, low cost, recyclability, and so on. Therefore, the composites have a wide range of applications in aerospace, automobile and motorcycle, office furniture, building materials, chemical industry, packaging engineering, and other industries. Therefore, it has been studied by many researchers. The glass fiber/polypropylene (GF/PP) composite properties have been improved using nanoplatelets [1,2]. The researchers examined the properties of GF/PP composite foaming materials [3,4]. The fatigue damage mechanism of GF/PP composites was studied by investigators [5]. Ku-Hyun Jung et al. developed a damage model with the interlaminar fracture toughness of GF/PP composites, and it accurately predicted the impact behavior of the materials [6]. The researchers enhanced the properties of GF/PP composites by using a β-nucleating agent [7,8,9]. Wu Deshun and Tang Ronghua et al. studied the properties of long glass fiber reinforced polypropylene composites [10,11]. Wang Xuanlun et al. found that the improvement of the interfacial bonding strength could significantly improve the properties of GF/PP composites [12]. The flame-retardant properties of GF/PP composites have been investigated [1,13]. The structure and interfacial shear strength of polypropylene/glass fiber/carbon fiber composites were studied using direct injection molding [14]. Furthermore, the researchers revealed the mechanisms of intrinsic toughening and extrinsic toughening of GF/PP composites [15].

The properties of glass fiber reinforced polyolefin composites are determined by the performances of glass fiber, a polymer matrix, and the interface between them. The interfacial adhesion between the fiber and resin matrix is critical to the properties of the composites to transfer stress through the interface, thus the interface shear strength determines the potential applications of whole composite materials. However, the surface of glass fiber is smooth with a low surface energy, and the wettability to resin is poor. At the same time, the glass fiber is polar, and with a few exceptions, the polymer matrix is generally non-polar, which leads to a vast polarity difference between them. The above reasons lead to the interface adhesion between the glass fiber and resin matrix not being ideal. Furthermore, it restricts the overall performance of composites. To achieve excellent total properties of composites, the adhesion and compatibility between the fiber and resin matrix are require improvement. Therefore, a lot of research has been done in this area. Zhang Daohai et al. enhanced the mechanical properties of long glass fiber reinforced polypropylene composites using grafted polypropylene with glycidyl methacrylate [16]. The performance of GF/PP composites was improved by grafting PP with acrylic acid [17]. Tang Ke et al. used maleic anhydride functionalized polypropylene (PP-G-MAH) to effectively enhance the interface adhesion between the continuous glass fiber and polypropylene resin, which resulted in excellent overall properties for the composites [18]. The performances of GF/PP and PP-banana/GF composites were improved using PP-G-MAH too [9,19,20]. Li Min et al. used polypropylene graft maleic anhydride and ethylene/octene copolymer to significantly improve the tensile, bending, and impact properties of GF/PP composites [21]. Research showed that the interface adhesion between GF and PP affected the GF/PP composite’s properties [22].

It was seen that these studies mainly focused on glass fiber reinforced polypropylene (GF/PP) composites by adding or subtracting or changing the composition of composites. There are few reports studied on the glass fiber reinforced polyethylene/polypropylene blends and their products. In particular, the influence of molding conditions on the structure and properties of glass fiber reinforced polyethylene/polypropylene composite pipes have rarely been reported on.

However, for the thin-walled tube under hydrostatic pressure, which was investigated in this paper, its circumferential stress and axial stress were calculated by Bai et al. [23] as follows:

(1) $$\sigma_{\theta }=\frac{pD}{2t}$$

(2) $$\sigma_{\alpha }=\frac{pD}{4t}$$

where σ_θ_ is the circumferential stress, σ_α_ is the axial stress, *t* is the pipe wall thickness, *D* is the pipe diameter, and *p* is the inside pressure. In this case, the relationship between σ_θ_ and σ_α_ is given in Equation (3):

(3) $$\sigma_{\theta }=2\sigma_{\alpha }$$

Therefore, for the thin-walled tube under hydrostatic pressure, its circumferential strength should be two times its axial strength to achieve the balance of pipe properties. However, many studies have shown that in the molding process, polymer macromolecules will be arranged and distributed along the flow direction of the melt to form the oriented structure along the route, thus enhancing the strength performance of the product along the path [24,25,26]. This phenomenon is evident during the formation of the oriented structure of GF too [27]. As a result, the circumferential strength of the pipe made using traditional manufacturing methods is far lower than its axial strength, and it is difficult to satisfy the practical demand for higher circumferential strength of the thin-walled tube under hydrostatic pressure. Although there have been many reports in the research of polymer pipe [28,29,30,31,32,33,34,35,36,37,38,39,40,41,42], these studies have not been able to effectively realize the self-enhancement of the pipe’s circumferential strength without reducing its axial strength, especially the coincident self-enhancement of the pipe’s axial and circumferential strength.

When the melt of GF-reinforced polymer composites is induced by an applied stress, the GF and macromolecule of the polymer under the induction of the stress will be arranged and distributed along the stress direction to form an oriented structure [43]. Furthermore, the oriented structure will self-improve the mechanical properties in the path. Inspired by this, in this study, a shearing–drawing two-dimensional compound stress field pipe-extrusion device was used, which could generate the applied stress along the circumferential and axial direction of the pipe at the same time, and the SGF/HDPE/PP composite pipes with better self-enhanced circumferential strength and self-enhanced axial strength were manufactured using it. The axial and circumferential strength of the tube and its structure and properties were comparatively investigated after the rotating shear sleeve of the device was stationary and rotated. By adjusting the rotating speed of the sleeve in the process of manufacturing, it can change the intensity of the applied shear stress along the circumferential direction of the pipe so its influence on the structure and performances of the tube can be studied. In this way, the circumferential and axial strength of the canal were self-enhanced simultaneously on the premise that no pipe raw materials were added, no raw material composition was changed, and no structure, shape, or size of the tube was changed. In particular, the circumferential strength of the pipe was significantly self-improved. Thus, the results of this experiment better optimized the material properties and better met the actual demand for material properties of a thin-walled tube under hydrostatic pressure.

## 2. Experimental

### 2.1. Materials

The high-density polyethylene (HDPE) used in this research was type 6100 M and purchased from Beijing Yanshan Petrochemical Co., Ltd. (Beijing, China). Its melting index was 0.42 g/10 min. The 30% short glass fiber reinforced polypropylene (SGFRPP) used in this research was granular material and purchased from Chemical Industry Zhonghao Chenguang Research Institute (Chengdu, China). The mass ratio of innovative raw materials in this study was as follows: HDPE/SGFRPP = 60/40.

### 2.2. The Process of Pipe Extrusion

**Figure 1:**
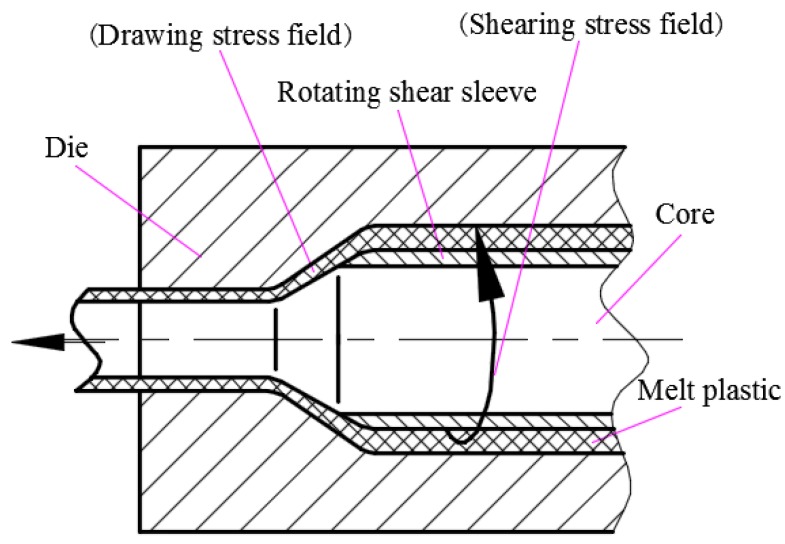
Schematic of the structure and principle of shearing–drawing two-dimensional compound stress field pipe-extrusion.

The pipe extrusion device was developed with an inner diameter of 30 mm and an outer diameter of 35 mm. The melt passed through the circumferential shear stress field and axial tensile stress field successively during extrusion. The rotating of the rotating shear sleeve on the mandrel generated the shear stress field on the material along the circumferential direction of the pipe, and the rotating speed could be adjusted. The reduction of the circular runner section size from the shear stress field section to the die section caused an axial drawing stress field along the axial direction on the material. When the composite melt flowed through the two stress fields, it was subjected to circumferential shear and axial drawing action successively, and then formed into the pipe through the extrusion die. The schematic diagram of the extrusion device is shown in Figure 1 [44].

From the feeding hopper to the die, the extrusion temperatures of the plastic extruder (SJ-45B, Shanghai Extrusion Machinery Factory Co., Ltd., Shanghai, China) successively were 100, 180, 210, 200, and 180 °C. The temperature of the shear stress field zone was 200 °C. The temperature of the extrusion die zone was 170 °C. The rotating speed of the extruder screw was 20 revolutions per minute (rpm). The rotating speed of the rotating shear sleeve varied from 0 to 25 rpm. When the rotating speed was zero, the extruded pipe was defined as the conventional pipe. When the shear rotary sleeve was rotating, the molded tube was called the reinforced pipe. When the tube was extruded away from the die, it was immediately quenched and cooled to finalize the pipe shape in room temperature water.

### 2.3. Characterization of Pipe

Thin slices of 0.3 mm thickness were cut from the core layer at the location of 1.1 mm away from the surface of the pipe. Under nitrogen protection, the second heating melting behavior was measured using a differential scanning calorimeter (DSC, TA2000, TA Instruments, Chicago, IL, USA) at temperatures ranging from room temperature to 200 °C at a rate of 10 °C/min, and then a delay of more than 3 min at the temperature of 200 °C. The following Equation (4) was used to calculate the crystallinities of PE and PP in the pipe [45].

(4) $$\alpha_{c}=\frac{\Delta H_{f}}{\Delta H_{f}^{0}}\times 100\%$$

where α_c_ is the crystallinity, ΔH_f_ is the latent heat of crystallization melting, and $\Delta H_{f}^{0}$ is the melt latent heat of the same polymer with the crystallinity of 100%. In this research, the value of PE’s $\Delta H_{f}^{0}$ was specified as 293 J/g, and the value of PP’s $\Delta H_{f}^{0}$ was defined as 209 J/g.

The crystal diffraction of pipe was analyzed using a polycrystalline X-ray Diffractometer (WAXD, D/MAX-Ⅲ, Rigaku Corporation, Akishima, Tokyo, Japan) under the conditions that the scanning speed was 0.06°/s, the voltage was 45 kV, and the pipe current was 40 mA. The 10 mm × 10 mm samples with entire wall thickness were directly cut from the pipe sample, and their axial directions were marked to distinguish between the axial and circular direction. Then, they were ground flat on both sides for testing.

The microstructure of pipe was observed using scanning electron microscopy (SEM, X-650, Hitachi Co., Ltd., Tokyo, Japan). The rectangular block samples of 5 mm × 10 mm were cut from the middle of the pipe wall. Then, the samples were roughly ground to flat with sandpaper along the thickness direction. After being refined and polished, the polished surface was soaked in a mixture etching solution of KMnO_4_, H_3_PO_4,_ and H_2_SO_4_ for 24 h. Finally, the samples were cleaned successively with 30% of H_2_O_2_, deionized water, and acetone, and dried. The temperature was not more than 45 °C in the process of all operations, and the samples were sputter-coated with gold before the SEM observations.

### 2.4. Tensile Strength Testing of Pipe

**Figure 2:**
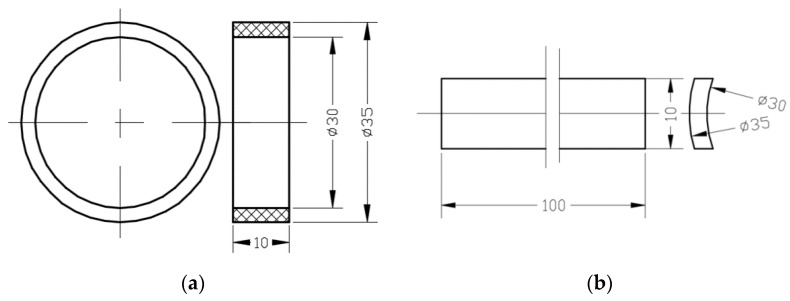
Schematic of the specimen for (**a**) circular tensile strength test and (**b**) axial tensile strength test.

**Figure 3:**
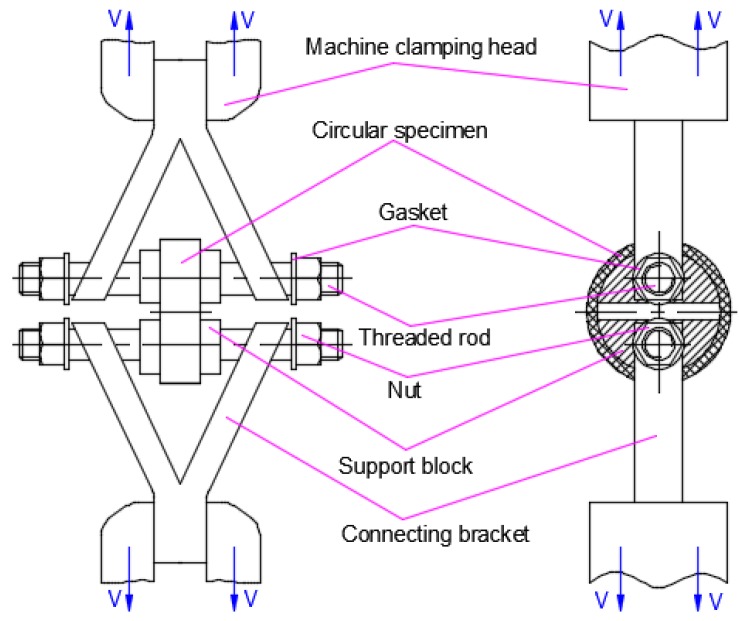
The scheme of loading for the circular tensile strength test.

The tensile strength of the pipe was tested using a universal electronic tensile testing machine (AG-10TA, Shimadzu Co., Ltd., Tokyo, Japan). The stretching velocity during the test was a constant 100 mm/min. The specimen for the circumferential tensile strength test was a circular specimen cut from the pipe samples. Its shape and sizes are exhibited in Figure 2a. The sample for the axial tensile strength test was taken as a straight strip specimen. Its form and dimensions are shown in Figure 2b [44]. The two ends of the straight strip specimen were directly clamped on the upper and lower clamping heads of the machine respectively for the axial tensile strength test. The circular sample was installed on the support blocks, and the support blocks were connected to the connecting brackets through the threaded rods. The connecting brackets were clamped on the upper and lower clamping heads of the machine for the circumferential tensile strength test. The loading scheme for circular tensile strength test is shown in Figure 3.

## 3. Results and Discussions

### 3.1. Orientation

**Figure 4:**
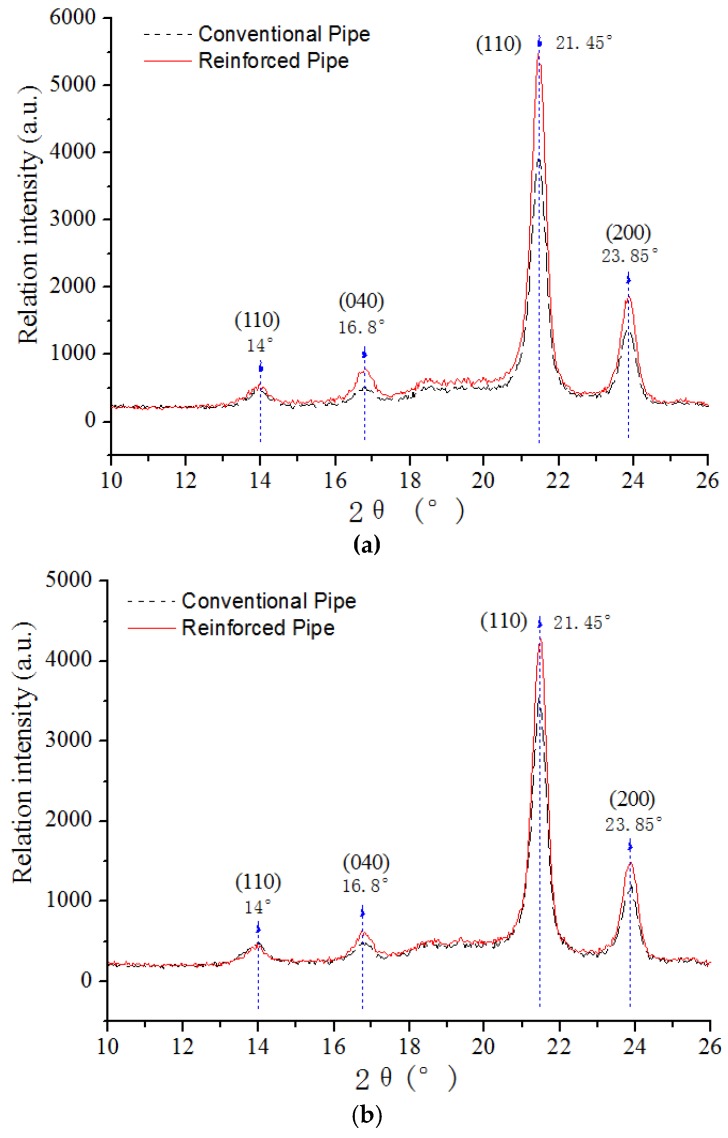
WAXD curves for the (**a**) circular direction and (**b**) axial direction.

WAXD characterization was carried out along the axial and circumferential directions of the conventional pipe and reinforced pipe with a rotating speed of 15 rpm. The results in Figure 4 show that the diffraction intensity of the {110} crystal plane at about 14° was almost the same as that of the conventional and reinforced pipe in both the circumferential and axial directions. However, the diffraction intensities of the {040} crystal plane at about 16.8°, {110} at approximately 21.45°, and {200} at approximately 23.85° of the reinforced pipe were significantly stronger than those of the conventional tube in both the circumferential and axial directions, indicating that the crystal orientation of the reinforced canal was higher than that of the traditional one. Furthermore, the diffraction intensities of all crystal planes of the reinforced pipe in its circumferential direction were higher than those in its axial direction. The results indicate that the introduction of shear rotating inducing a rotating shear sleeve induced the molecule structure of SGF/HDPE/PP pipe to obtain a better orientation effect along the circumferential direction, and thus the orientation degree along the route was enhanced. The formation of the more oriented structure along the circumferential direction of the pipe was conducive to self-improving the circumferential strength, promoting the reasonable distribution of the pipe properties and the effective utilization of the materials.

### 3.2. Heat Behavior

**Figure 5:**
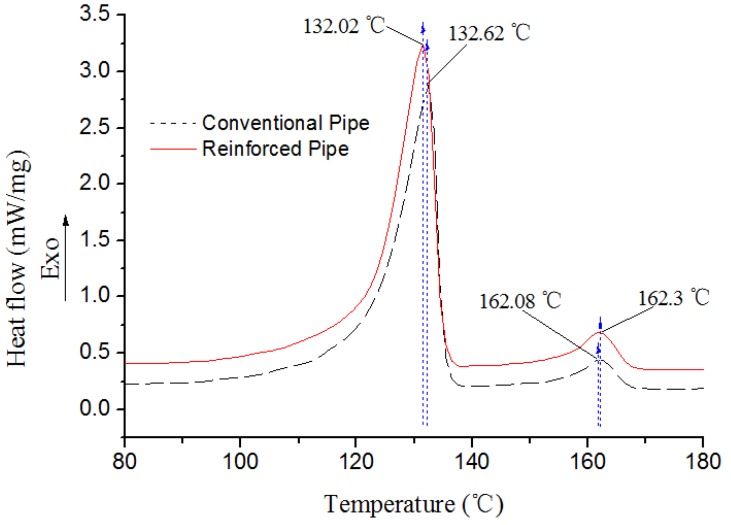
The second heating DSC curves for the SGF/HDPE/PP pipe.

The DSC analysis test of the conventional pipe and reinforced pipe with a rotating speed of 15 rpm was carried out, and the results are shown in Figure 5 and Table 1. The results in Figure 5 show that the melting peaks (*T_m_*) of PE and PP in the reinforced pipe were almost the same as those of the conventional one, as indicated by the results of *T_m_* in Table 1, meaning that their crystal plate thickness was virtually equivalent. In Table 1, the results also show that the melting ranges (Δ*T*) of PE and PP in the reinforced pipe were nearly the same as those of the conventional one too, indicating that their crystal plate thickness uniformity and crystallization rate were virtually the same as each other. However, as shown in Table 1, the PE’s crystallinity (α*_c_*) in the reinforced pipe increased from 54.91% in the conventional tube to 57.68%, a proportional increase of 5.04%. The α*_c_* of PP increased from 6.07% to 8.09%, a proportional increase of 33.28%. The results indicate that the rotation of the rotating shear sleeve formed an effective shearing-induced crystallization effect, which accelerated the formation of molecular crystallization of the SGF/HDPE/PP system and improved the crystallization effect of PP especially. The reason for the increases is believed to be that the rotation of the rotating shear sleeve induced the SGF/HDPE/PP system molecules to form a more orderly arrangement structure, especially along the circumferential direction of the pipe. This ordered structure promoted the crystallization of the system molecules. Then, the improvement of crystallinity was helpful to self-improve the mechanical properties of the tube.

### 3.3. Microstructure

**Figure 6:**
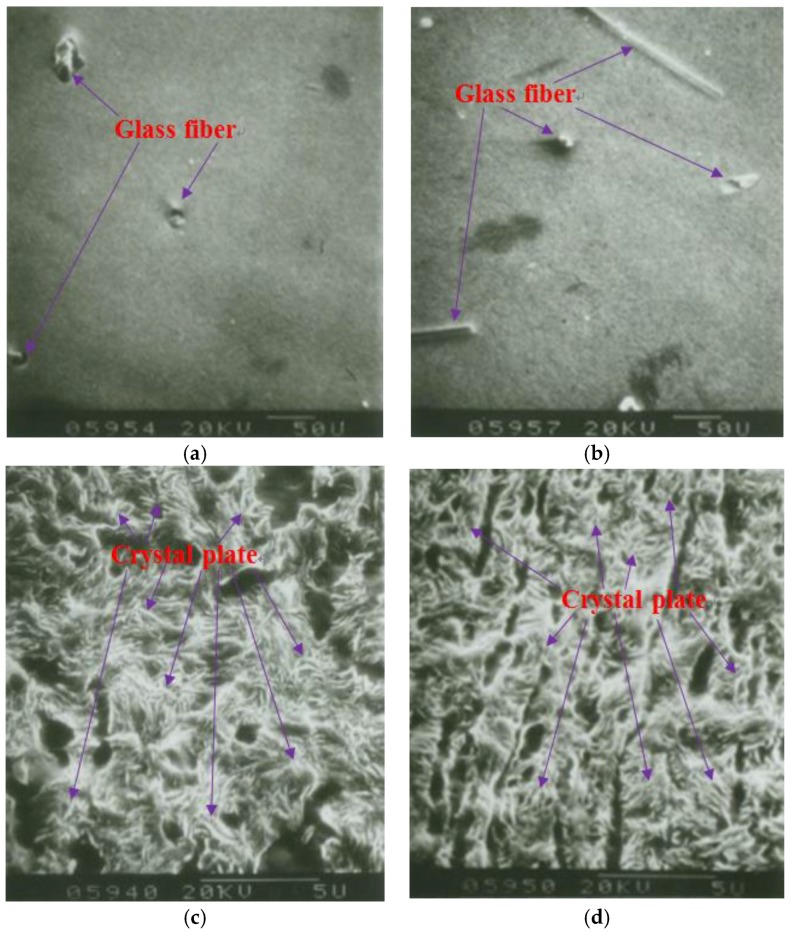
SEM micrographs of (**a**,**c**) conventional pipe and (**b**,**d**) reinforced pipe with 15 rpm.

The SGFs and crystal chips in the pipe were observed using SEM. The results can be seen in Figure 6a that there were few SGFs in the conventional tube arranged and distributed along the circumferential direction. However, the SGFs in the reinforced pipe with the rotating speed of 15 rpm was distributed and oriented along the circumferential path, as seen in Figure 6b. The results indicate that when the rotating shear sleeve rotated, it induced the SGFs to form an oriented distribution structure along the circumferential route of the pipe. The formation of the oriented structure of SGFs effectively self-enhanced the pipe properties. Furthermore, the results in Figure 6c show that the crystal plate in the conventional pipe was less deformed and oriented and it was randomly distributed. Compared with the results in the traditional tube, the crystal plate size in the reinforced canal was refined and became more uniform. Furthermore, the crystal plate was stretched and deformed to form an oriented structure along the circumferential direction of the tube, as shown in Figure 6d. The results indicate that the rotation of the rotating shear sleeve promoted the uniform structure and refinement of the crystal grain of SGF/HDPE/PP pipe and induced the orientation of the molecule along the circumferential direction. Furthermore, it self-improved the pipe’s strength performance, mainly the circumferential strength performance.

### 3.4. Tensile Strength

**Figure 7:**
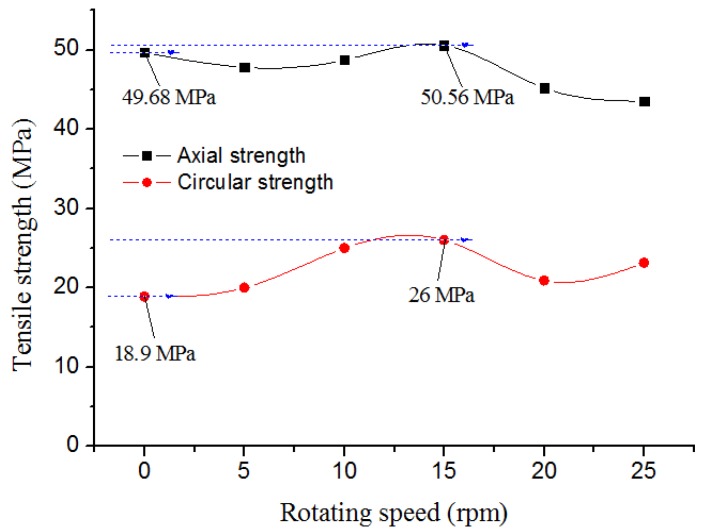
The tensile strength of the SGF/HDPE/PP pipe.

To investigate the tensile strength of the conventional and reinforced pipe, the axial and circumferential tensile strength tests were carried out. As seen in Figure 7, the axial and circumferential tensile strength of SGF/HDPE/PP pipe was self-enhanced at the same time when the rotating speed of the rotating shear sleeve was increased from 0 to 15 rpm. The axial tensile strength increased from 49.68 MPa for the conventional pipe to 50.56 MPa for the most muscular reinforced tube, a proportional increase of 1.77%. At the same time, the circumferential tensile strength increased from 18.9 MPa for the conventional pipe to 26 MPa for the most robust reinforced tube, a proportional increase of 37.57%. The results indicate that the rotation of the rotating shear sleeve could self-improve the axial and circumferential tensile strength performance of the pipe simultaneously, and in particular, the circumferential tensile strength performance self-improved significantly. This further optimized the performance of the composite materials and more fully satisfied the practical needs of the use of the thin-walled pipe under hydrostatic pressure. This may be because the shear stress field increased the order of SGFs and molecules of the tube, resulting in the oriented and distributed structure, in particular, the circumferential oriented and distributed architecture. However, the results in Figure 7 also show that the axial and circumferential tensile strength of SGF/HDPE/PP pipe decreased when the rotating speed exceeded 15 rpm. This indicates that when the sleeve rotated too violently, the axial and circumferential tensile strength of the pipe decreased. The reason could be that the adverse effect of the disorientation and recovery of the molecule and SGF, which was caused by the shear heat generated by the sharp rotating of the sleeve, was greater than the positive effect of the order enhancement of the SGFs and molecules induced by the shear stress field.

## 4. Conclusions

In this study, SGF/HDPE/PP composite pipes were extruded through a shearing–drawing two-dimensional compound stress field pipe-extrusion device, with the aim to self-improve the circular and axial properties of the tube at the same time. The change of the structure and performance of the pipe after the shear rotary sleeve was stationary and rotated was studied comparatively. The results show that the circumferential and axial strength of the tube could be self-enhanced synchronously via the rotation of the rotating shear sleeve, and in particular, the effect of the circumferential strength self-reinforcement was clear. The axial tensile strength increased from 49.68 MPa for the conventional pipe up to 50.56 MPa for the most robust reinforced tube, a proportional increase of 1.77%. The circumferential tensile strength increased from 18.9 MPa for the traditional pipe to 26 MPa for the most robust reinforced one, a proportional increase of 37.57%. The SEM results confirmed that the crystal and SGF in the reinforced tube had been oriented along the circumferential and axial direction simultaneously. The results of WAXD demonstrated that the orientation degree of the reinforced tube was higher than that of the conventional one, and it had a better orientation effect along the circumferential direction than that along the axial route in the reinforced tube. The results from the DSC analysis showed that the crystallinity of the reinforced pipe was improved and the crystal grain was well refined. As a result, the crystallinity of PE increased from 54.91% in the conventional tube to 57.68% in the reinforced one, a proportional increase of 5.04%. The crystallinity of PP rose from 6.07% in the traditional pipe to 8.09% in the reinforced one, a proportional increase of 33.28%. In this way, the properties of the circumferential and axial strength of the SGF/HDPE/PP composite pipe were self-improved concurrently, and it fully meets the functional requirements for the use of the thin-walled tube under hydrostatic pressure.

## Figures and Tables

**Table 1:** The second heating DSC testing results of the SGF/HDPE/PP pipe.

Pipe Type	Polymer Type	ΔH_f_ (J/g)	*T*_m_ (°C)	Δ*T* (°C)	α_c_ (%)
Reinforced	PE	169	132.02	12.40	57.68
	PP	16.9	162.30	10.79	8.09
Conventional	PE	160.9	132.62	12.49	54.91
	PP	12.68	162.08	10.62	6.07
